# Pathological application of carbocyanine dye-based multicolour imaging of vasculature and associated structures

**DOI:** 10.1038/s41598-020-69394-0

**Published:** 2020-07-28

**Authors:** Alu Konno, Naoya Matsumoto, Yasuko Tomono, Shigetoshi Okazaki

**Affiliations:** 1 Institute for Medical Photonics Research, Preeminent Medical Photonics Education and Research Center, Hamamatsu University School of Medicine, Hamamatsu, Japan; 2 Central Research Laboratory, Hamamatsu Photonics K.K., Hamamatsu, Japan; 3 Division of Molecular and Cell Biology, Shigei Medical Research Institute, Okayama, Japan

**Keywords:** Imaging, Fluorescence imaging, Microscopy, Confocal microscopy, Multiphoton microscopy, Glomerular diseases, IgA nephropathy, Kidney

## Abstract

Simultaneous visualisation of vasculature and surrounding tissue structures is essential for a better understanding of vascular pathologies. In this work, we describe a histochemical strategy for three-dimensional, multicolour imaging of vasculature and associated structures, using a carbocyanine dye-based technique, vessel painting. We developed a series of applications to allow the combination of vessel painting with other histochemical methods, including immunostaining and tissue clearing for confocal and two-photon microscopies. We also introduced a two-photon microscopy setup that incorporates an aberration correction system to correct aberrations caused by the mismatch of refractive indices between samples and immersion mediums, for higher-quality images of intact tissue structures. Finally, we demonstrate the practical utility of our approach by visualising fine pathological alterations to the renal glomeruli of IgA nephropathy model mice in unprecedented detail. The technical advancements should enhance the versatility of vessel painting, offering rapid and cost-effective methods for vascular pathologies.

## Introduction

Blood vessels form a network that delivers molecules and cells throughout the body. Because of their three-dimensional (3D) nature, it is difficult, or at least painstaking, to understand their distribution by observing conventional histological sections. Recent advancements in optical sectioning microscopy, such as confocal, multi-photon, and light-sheet microscopies, have made volume imaging of vasculature much easier. These fluorescence-based microscopies require effective and specific labelling techniques for imaging vasculature in 3D specimens. These techniques include genetically encoded fluorescent proteins, specific probes such as antibodies and lectins, or infusion of space-occupying materials^1–4^. Vessel painting is a method of labelling the entire vasculature of small mammals through perfusion of a lipophilic carbocyanine dye, DiI (1,1′-dioctadecyl-3,3,3′,3′-tetramethylindocarbocyanine)^5–9^. Recently, we improved labelling intensity and uniformity, as well as the reproducibility of the technique, by introducing a neutral liposome and a hydrophilic DiI analogue^10^.

Although vessel painting is an easy and cost-effective technique to label vasculature intensely, it has several limitations. First, the excitation/emission spectra of DiI largely overlap with popular red fluorophores, such as Alexa 568 and mCherry, which limits the combination of probes for multi-labelling. Second, liposome-mediated vessel painting has only been applied to the vasculature of the central nervous system (CNS) of small rodents and its applicability to other organs needs to be tested^5–9^. Third, its compatibility with various tissue clearing protocols has not been tested, with the exception of an expensive proprietary reagent of unknown composition, FocusClear^7^. Finally, its compatibility for multi-labelling with other types of probes, such as antibodies or fluorescently labelled small molecules, except for nuclear staining^10^, has not been carried out.

In this study, we aimed to extend the utility of vessel painting by overcoming the limitations mentioned above. We tested several commercially available DiI analogues and successfully introduced three additional dyes with green (DiO), deep red (DiD), and near-infrared (DiR) fluorescence to this technique to increase the number of choices for available colours. Then, we confirmed that the liposome-mediated vessel painting is applicable for imaging the vasculature of various non-CNS organs. We also sought tissue clearing protocols that are compatible with vessel painting. In this exploration, we also developed a novel aberration-correction technique that makes a single objective compatible with immersion fluids with a broad range of refractive indices (RIs), from water to tissue clearing reagents with high RIs. This novel technique improves the image quality deteriorated by the RI mismatch between an objective and an immersion medium through wavefront control of the incident light using a spatial light modulator (SLM)^11,12^ that is incorporated within our two-photon microscope. Then, we selected the mouse kidney as a model organ to demonstrate the versatility of vessel painting combined with various histochemical techniques. To this end, we developed a thin-sectioning-free and rapid multi-labelling method for 3D visualisation of renal structures, especially glomeruli. Finally, we combined vessel painting with a tissue clearing protocol and other labelling methods to image pathological changes of the glomeruli of hyper-IgA (HIGA) mice, an IgA nephropathy model strain^13–15^. Confocal and two-photon microscopies of the multi-labelled glomeruli from HIGA mice successfully visualised minute lesions that had only been observed with transmission electron microscopy previously. With the novel aberration-correction technique, it was demonstrated that two-photon microscopy could visualise entire glomeruli at subcellular resolution in renal specimens and the alteration of podocyte distributions in HIGA mice.

## Results

DiI and its analogues are hydrophobic carbocyanine dyes that can label a plasma membrane by inserting alkyl chains into the lipid bilayer. In liposome-mediated vessel painting, carbocyanine dye molecules are first inserted into the liposomal membrane and the liposomes fuse with the plasma membrane upon perfusion (Supplementary Fig. S1). The length of the alkyl chains of DiI is an important factor for the quality of vessel painting^10^. Therefore, for clarity, we designate the number of carbons in the alkyl chains in parentheses after the name of the dye [e.g., DiI(C12) and DiI(C18) for DiIs with 12- and 18-carbon-long alkyl chains, respectively].

### The introduction of new carbocyanine dyes to vessel painting provides more colour choices

Currently, DiI(C12) has been the only carbocyanine dye used for liposome-mediated vessel painting. The use of other DiI analogues with different fluorescence properties for this technique could be convenient. Therefore, we tested commercially available DiI analogues with green [DiO(C14), DiO(C18), and Neuro-DiO(C18)], deep red [DiD(C18)], and near-infrared [DiR(C18)] fluorescence for liposome-mediated vessel painting (the structure of the dyes tested are shown in Supplementary Fig S2). DiO(C14), Neuro-DiO(C18), DiD(C18), and DiR(C18) were readily soluble in ethanol at a concentration of 5 mM. We excluded DiO(C18) from our experiments because of its poor solubility in ethanol.

**Figure 1:**
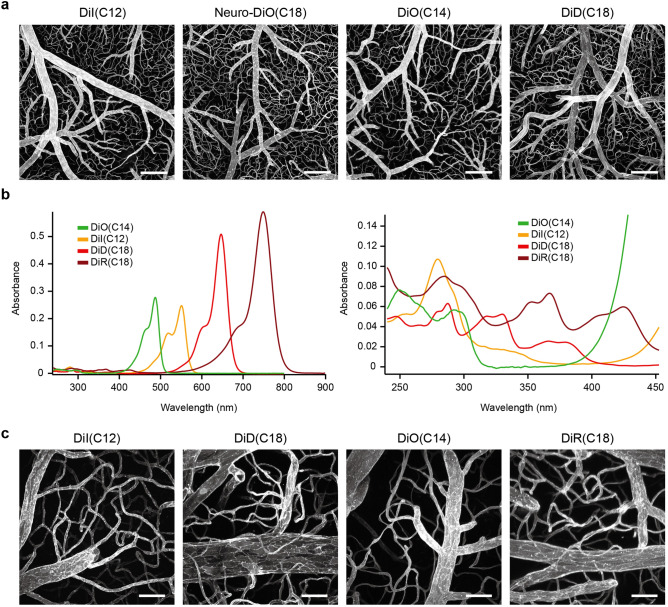
The introduction of DiI analogues to liposome-mediated vessel painting provides greater wavelength flexibility. (**a**) Representative confocal images of cerebral vasculature labelled by liposome-mediated vessel-painting. Maximum projections of optical sections from the brain surface (*N* = 1 for Neuro-DiO(C14) and *N* = 2 for other dyes; each image is a representative of more than three observations). (**b**) Absorption spectra of DiI and its analogues. The right panel is spectrum of the shorter wavelength region of the spectrum in the left panel showing. These measurements were performed using solutions of 2 μM (left panel) and 10 μM (right panel) in ethanol. (**c**) Representative two-photon microscopy of the cerebral vasculature. Maximum projections of optical sections from the brain surface (*N* = 1 for each dye; each image is a representative of three observations). Scale bars = 200 μm (**a**) and 50 μm (**c**).

We then performed liposome-mediated vessel painting with DiO(C14), Neuro-DiO(C18), DiD(C18), as well as DiI(C12) and imaged cerebral vasculature with confocal microscopy (Fig. 1a). Although Neuro-DiO(C18) appeared to successfully label vasculature (Fig. 1a), it sometimes caused leakage of the perfusate from the airway. None of the other tested dyes caused such leakage.

Next, we tested the applicability of these dyes for two-photon microscopy. First, we measured the absorption spectra of the dyes to know the excitation wavelength ranges (Fig. 1b). In addition to the main peaks, small absorption bands were also found in the shorter wavelength region (Fig. 1b, right panel). We, thus, confirmed that DiO(C14), DiI(C12), DiD(C18), and DiR(C18) are available for two-photon microscopy (Fig. 1c), and it can be concluded that DiO(C14), DiD(C18), and DiR(C18), as well as DiI(C12), are suitable for reproducible liposome-mediated vessel painting.

### Vessel painting is applicable to various organs

**Figure 2:**
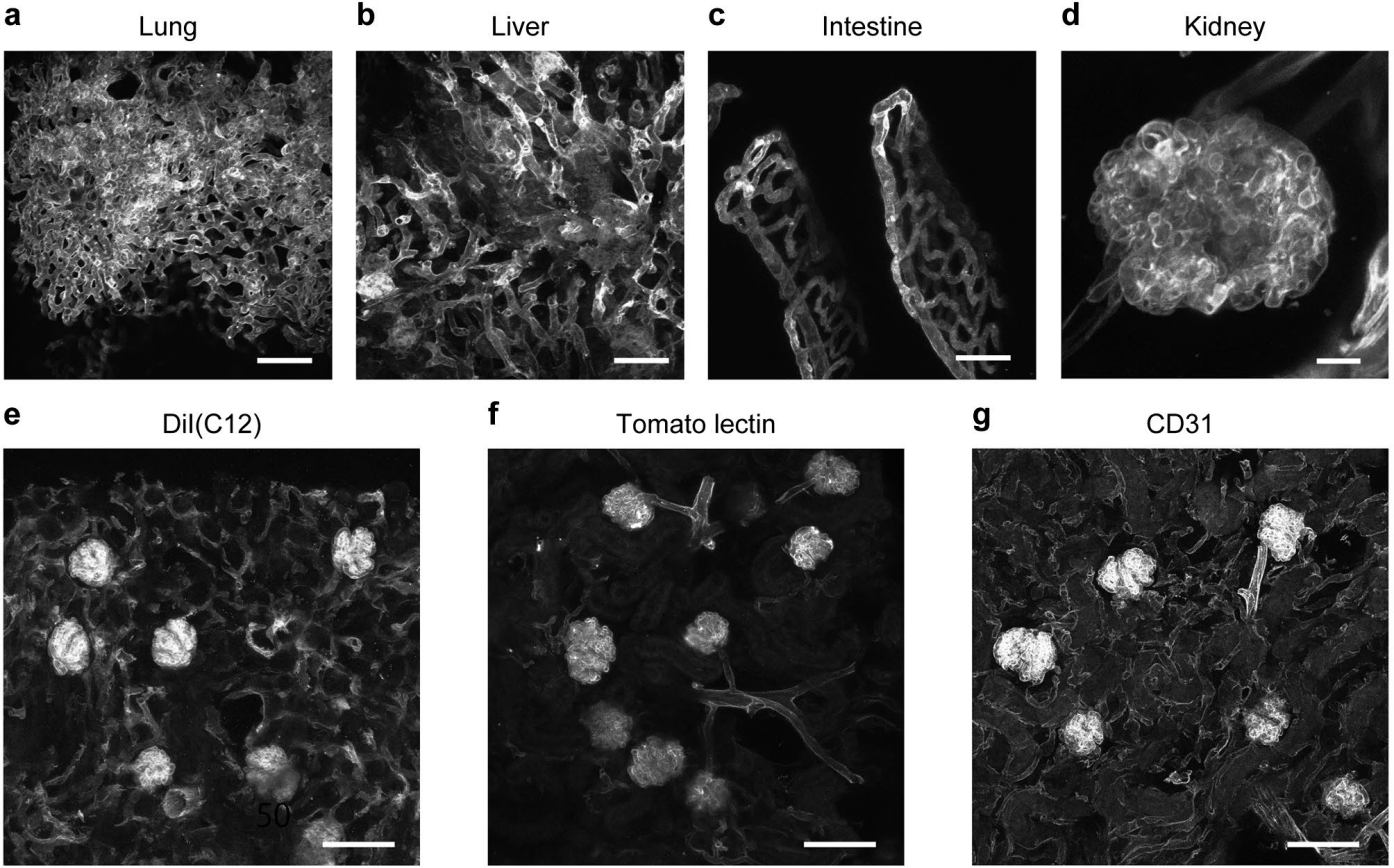
Vessel painting is applicable to various organs. (**a–d**) Two-photon microscopy of DiR(C18)-labelled blood vessels in lung (**a**), liver (**b**), intestine (**c**), and kidney (**d**) tissues. *XY* maximum projections of optical sections from sample surfaces (*N* = 2; representative images of more than six observations). Scale bars indicate 50 µm (**a–c**) and 10 µm (**d**). (**e–f**) Comparison of labelling patterns among vessel painting, fluorescently-labelled tomato lectin, and anti-CD31 antibody for renal vasculature. Representative confocal images of liposome-mediated vessel painting with DiI(C12) [*N* = 2 for DiI(C12) and tomato lectin, *N* = 1 for CD31; each image is a representative of more than five observations] (**e**), Tomato lectin (**f**), and anti-CD31 antibody (**g**). Maximum projections of optical sections from sample surfaces. Scale bar = 100 µm (**e–f**).

The application of liposome-mediated vessel painting has only been tested for the staining of CNS vasculature. We expected that its ligand-independent labelling principle allows visualisation of any type of blood vessel in various organs. Therefore, we performed two-photon microscopy of the lung, liver, intestine, and kidney dissected from mice after vessel painting (Fig. 2a–d). After the perfusion of DiR(C18), blood vessels, including capillaries, were intensely labelled in all the organs tested. We also compared the labelling pattern of liposome-mediated vessel painting with those of tomato lectin and anti-CD31 antibody for renal vasculature (Fig. 2e–g). We found that tomato lectin labelled far fewer blood vessels than vessel painting (Fig. 2e–g). The labelling patterns of vessel painting and intravenously injected anti-CD31 antibody were comparable. However, the anti-CD31 antibody and the lectin required much (~ 100 times) more laser power to obtain comparable signal intensity to vessel painting. Therefore, we concluded that liposome-mediated vessel painting is a reliable method to visualise vasculature in various organs and tissues.

### A dye with longer emission wavelength increases vasculature imaging depth

**Figure 3:**
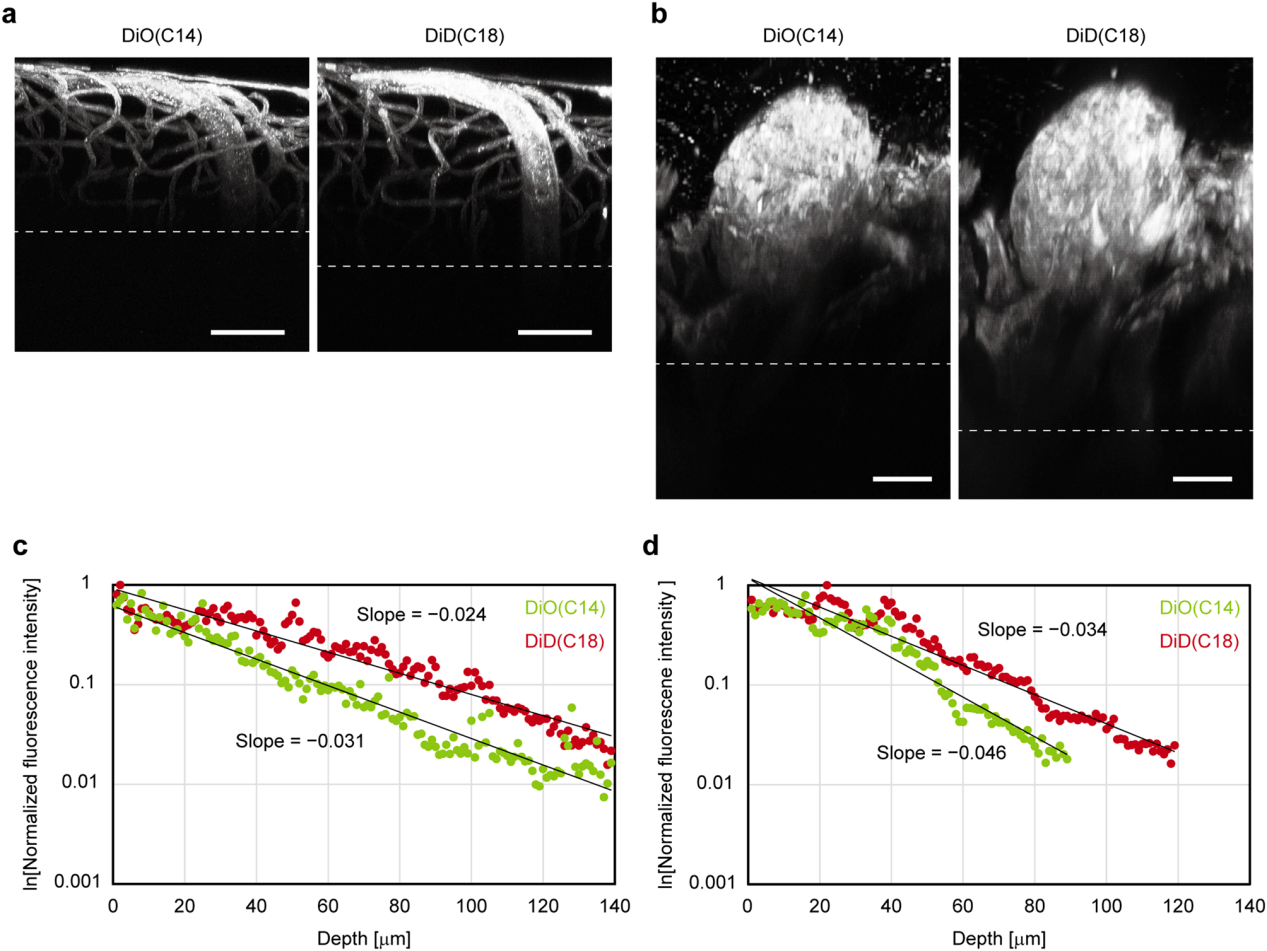
A dye with a longer emission wavelength increases the vasculature imaging depth. Representative two-photon microscopy *XZ* projection images of the brain (**a**) and kidney (**b**) double-vessel painting specimens labelled with DiO(C14) and DiD(C18) (*N* = 2 for each). Both dyes are simultaneously excited at 820 nm. Scale bars = 50 μm (**a**) and 20 μm (**b**). (**c**, **d**) Semi-logarithmic plots of normalised maximum fluorescence intensities for optical sections of brain (**c**) and kidney (**d**) tissue versus depth. Data from the optical sections shown in (**a**) and (**b**) are used to generate these plots. The lines are exponential fits of the fluorescence intensities and their slopes indicating the signal attenuation rates.

Generally, probes with longer emission wavelengths allow deeper imaging. In our study, we evaluated the effect of the emission spectra of the carbocyanine dyes on deep-tissue imaging by two-photon microscopy (Fig. 3). We found that DiI and its analogues have small absorption bands in the shorter spectral region in addition to their absorption maximum peaks (Fig. 1b). Therefore, when an appropriate excitation wavelength is selected, two different carbocyanine dyes are excited simultaneously, and the effects of excitation and emission wavelengths on imaging depth can be separated. We performed double vessel painting with DiO(C14) and DiD(C18) and compared the imaging depth attainable with each dye through two-photon microscopy of brain and renal tissues (Fig. 3a,b). We then plotted the normalised maximum fluorescence intensities of the optical sections from the date for Fig. 3a,b on semi-log graphs (Fig. 3c,d). In these plots, the slope of the straight line fitted to the data corresponds to the rate of the attenuation of the maximum fluorescence intensity with depth^16^: a less-steep slope is more favourable for deeper imaging. Therefore, these results indicate that DiD(C18) can improve imaging depth by approximately 20% compared with DiO(C14), for both brain and kidney tissue, under the same excitation wavelength condition.

### Vessel painting is compatible with tissue clearing protocols that are free from detergent or solvent

Next, we tested the combination of vessel painting and tissue clearing protocols for deeper imaging. Since lipophilic carbocyanine dyes get extracted along with lipids by organic solvents or detergents, we selected three tissue clearing protocols that are free from these reagents: SeeDB^17^, Sca*l*eSQ(0)^18^, and OPTIClear^19^. Although those protocols were originally developed for brain tissue, we also tested their clearing efficiency for kidney tissue.

In this experiment, we used a novel SLM-based technique to correct aberration that occurs at the interface of an objective and an immersion fluid. An SLM incorporated into the two-photon microscope modulates the incident light wavefront, cancelling the aberration caused by the mismatch of the recommended RI of an objective with the actual RI of the medium in which the sample is immersed (Supplementary Fig. S3). This technique is feedback-free and works if the RI of an immersion medium is known. With this technique, a single objective can be adopted for a broad RI range of immersion solutions. This is a desirable feature as it allows comparison of the clearing properties of different tissue clearing solutions with different RIs.

**Figure 4:**
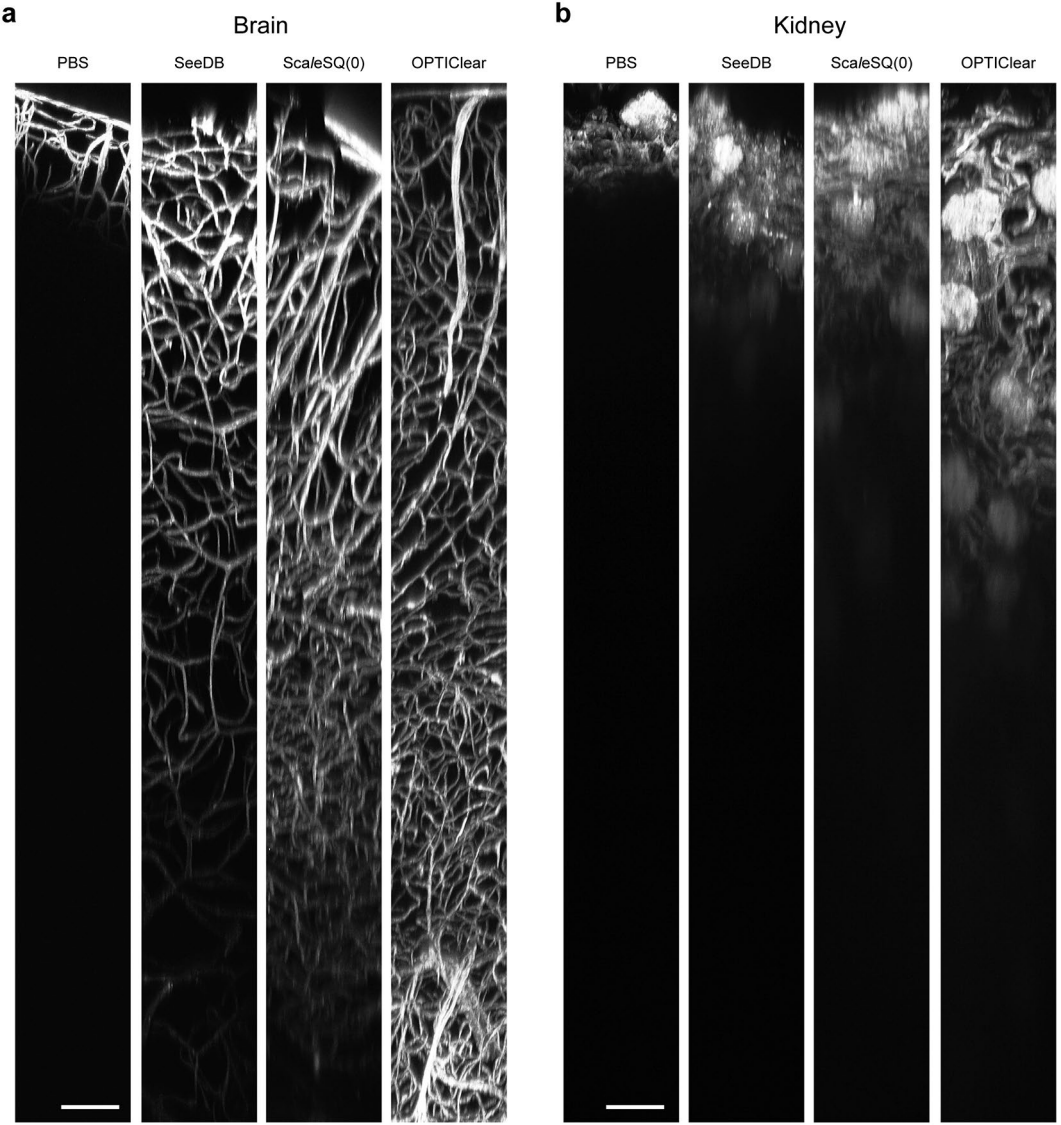
Vessel painting is compatible with tissue clearing protocols that are free from detergent or solvent. Two-photon microscopy *XZ* projection images of brain (**a**) and kidney (**b**) tissues cleared with SeeDB, Sca*l*eSQ(0), and OPTIClear after vessel painting with DiR(C18). (*N* = 3; each image is a representative of at least three observations). For each tissue type, the power of the laser, measured before the objective, is fixed at a value that generates similar signal intensities at the sample surface and kept constant.[Laser powers are as follows. (**a**) PBS 38.8 mW, SeeDB 48.7 mW, Sca*l*eSQ 63.4 mW, OPTIClear 48.7 mW. (**b**) PBS 27.5 mW, SeeDB 28.6 mW, Sca*l*e SQ 45.4 mW, OPTIClear 48.7 mW.] Scale bar = 100 μm. The vertical extent of each image corresponds to 1800 μm.

We performed two-photon microscopy for brain and kidney samples labelled with DiD(C18) and cleared with SeeDB, Sca*l*eSQ(0), or OPTIClear, as well as control samples in phosphate buffer saline (PBS). We confirmed that all three tissue clearing protocols are compatible with vessel painting and improve imaging depth in both brain and renal tissues (Fig. 4a,b). Among them, OPTIClear showed the strongest clearing capacity. OPTIClear-treated brain tissue did not show noticeable attenuation of signals even at depths of 1800 µm (Supplementary Fig. S4). Our aberration-correction technique allowed a single water immersion objective to be adjusted for the immersion fluids with different RIs, increasing the signal intensities and signal-to-noise ratios. We used this system for two-photon microscopy reported in this article.

### Application of vessel painting to renal pathological analysis

Next, we attempted to develop a multi-labelling method with various probes after vessel painting. Although carbocyanine dyes are incompatible with common permeabilisation treatment with detergents, we expected that fixed dead cells would be macromolecule permeable. We also assumed that a porous tissue is especially favourable for the diffusion of probes. Hence, we explored the possibility of permeabilisation-free post-fixation labelling with kidney slices.

**Figure 5:**
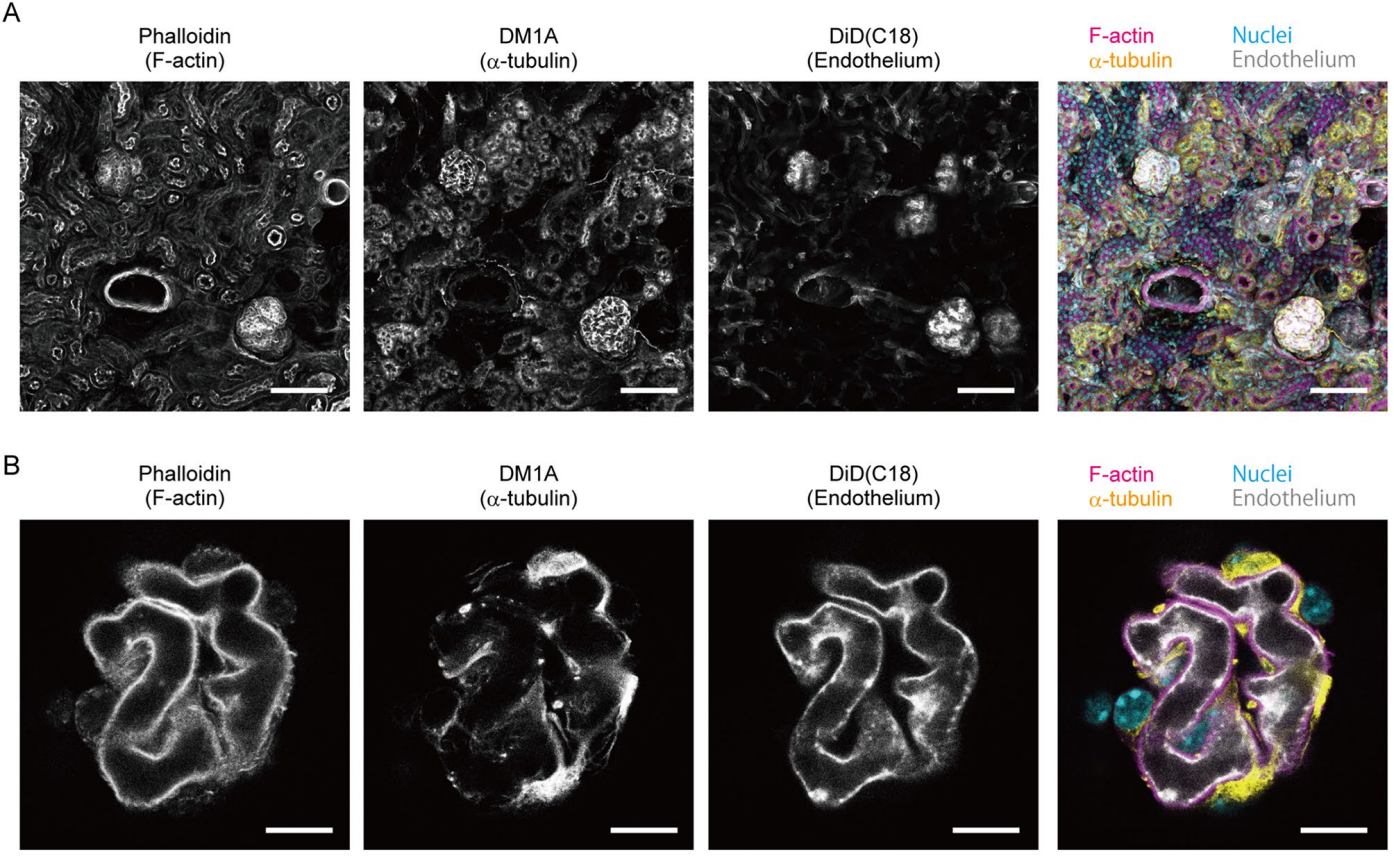
Glomeruli can be labelled with various probes after vessel painting. Confocal microscopy of quadruple-labelling of renal tissues multi-labelled with vessel painting and other probes. (**a**) *XY* maximum projection of confocal optical sections taken from the surface of a renal slice. (**b**) A confocal optical section of a glomerulus. F-actin (magenta), a-tubulin (yellow), endothelium (grey), and nuclei (cyan) are labelled with rhodamine-phalloidin, DM1A Alexa 488 conjugate, DiD(C18), and DAPI, respectively. (N = 2; each image is a representative of 19 observations.) Scale bars = 100 μm (**a**) and 10 μm (**b**).

We first performed preliminary experiments to test whether a small probe and an antibody can label 3D structures on renal slices without permeabilisation with a detergent (Supplementary Fig. S5). Both fluorescently labelled phalloidin and anti-α-tubulin antibody (DM1A) specifically labelled various renal structures exposed to the surface of slices without specific permeabilisation steps. Only 1 h incubation with these probes was sufficient to observe an entire glomerulus at the surface of a slice. The omission of thin sectioning allowed us to image intact structures of glomeruli and to complete the total procedure, from vessel painting to imaging, within one day. In glomeruli, the cell body/major processes and foot processes were nearly exclusively labelled with anti-α-tubulin antibody and phalloidin, respectively. Foot processes were visualised in some detail with a conventional diffraction-limited confocal microscope (Supplementary Fig. S5). Podocytes surrounded by intact Bowman’s capsule showed lower intensity signals, possibly due to poor penetration of probes (data not shown). Next, we performed confocal microscopy of glomeruli labelled with phalloidin, 4′,6-diamidino-2-phenylindole (DAPI), and anti-α-tubulin antibody after vessel painting. The quadruple-staining of renal slices simultaneously visualised intact 3D structures of glomerular capillary tufts, podocyte processes, and nuclei (Fig. 5). Therefore, multi-labelling with various probes after vessel painting is possible, at least for renal substructures exposed at the surface of tissue slices.

### Vessel painting is applicable for analysis of glomerular pathology

**Figure 6:**
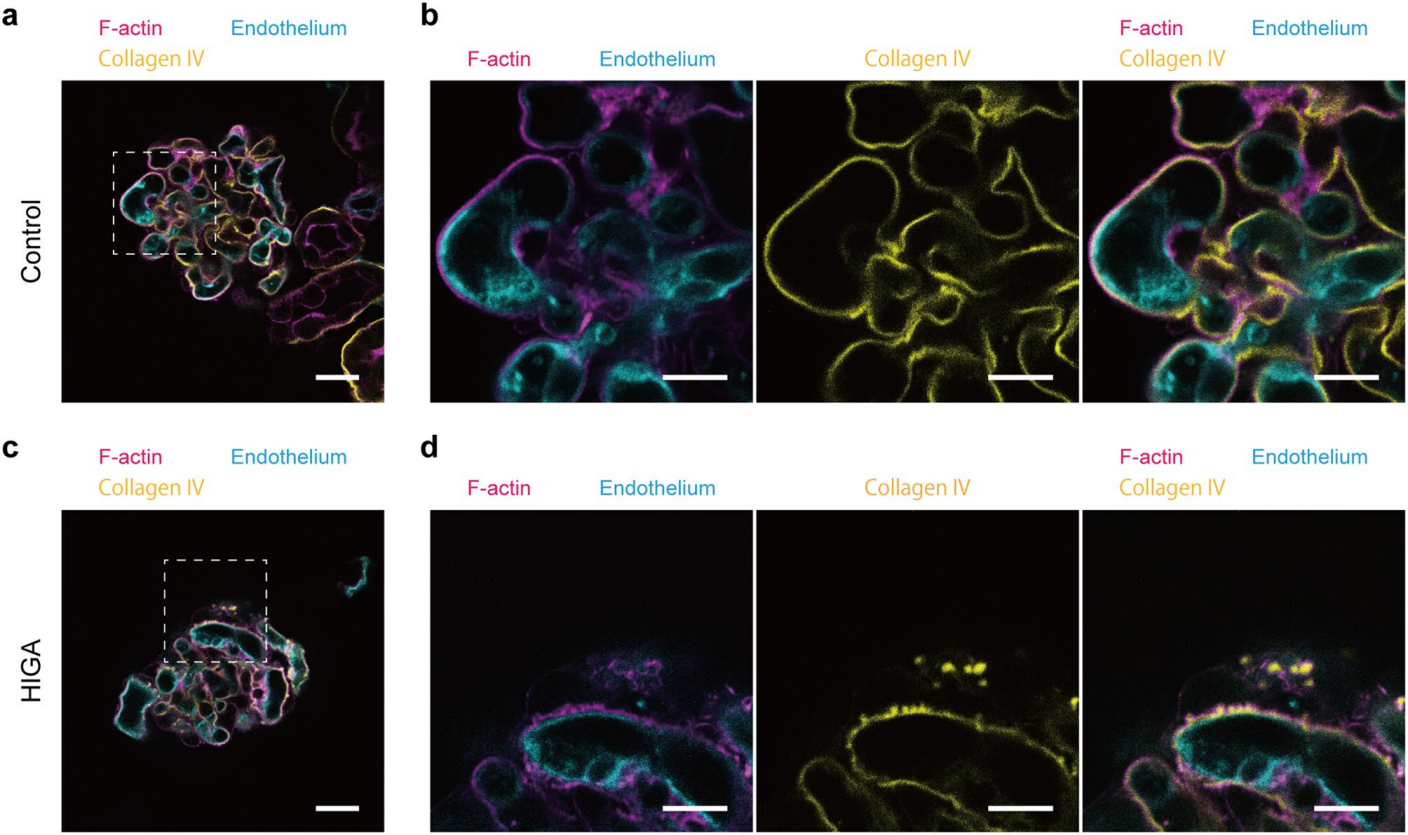
Vessel painting is applicable for the analysis of glomerular pathology. Two-photon microscopy of glomeruli from single HIGA (**c**, **d**) and age-matched control BALB/c (**a**, **b**) mice labelled by vessel painting with DiD(C18) (endothelium: cyan), phalloidin (foot processes: magenta), and anti-collagen IV antibody (GBM: yellow). Scale bars = 20 µm (**a**, **c**) and 10 µm (**b**, **d**). [*N* = 1; each image is a representative of 10 (control) or 3 (HIGA) measurements. The result from other individuals is in Supplementary Fig. S6].

As a proof-of-principle experiment to demonstrate the versatility of vessel painting by combining it with other histochemical methods, we performed imaging experiments on the glomeruli of HIGA mice, a model for IgA nephropathy^13–15^. Glomeruli on the surface of kidney slices from 25–30-week-old HIGA mice and age-matched control BALB/c mice were labelled by vessel painting with DiD(C18) and then triple-stained with DAPI, anti-α-tubulin antibody, and rhodamine-phalloidin to visualise nuclei, cell bodies and major processes of podocytes, and foot processes, respectively. Confocal microscopy of those quadruple-stained glomeruli revealed that the phalloidin-labelled foot processes of HIGA mice include numerous foam-like structures, known as glomerular basement membrane (GBM) nodules^13,15,20^ (Supplementary Fig. S6); although these were also observed in the normal control animals, the frequency was much lower. With anti-collagen IV suncus monoclonal antibody^21^, we confirmed that the GBM nodules were outwardly directed local thickening of the GBM (Fig. 6, Supplementary Fig. S6, Supplementary Movies S1 and S2). The GBM nodules were not discernible when the renal tissue blocks from HIGA mice were subjected to conventional histological processing (Supplementary Fig. S6). In addition, some podocytes of HIGA mice were labelled with the carbocyanine dye in 55.5% (10 out of 18) of the glomeruli observed, suggesting that liposomes penetrated through the ultrafiltration barrier. Similar staining patterns were not observed in the control mice (0 out of 20 observed glomeruli) (Supplementary Fig. S7).

### Glomeruli in HIGA mice show reduced major processes of podocytes

**Figure 7:**
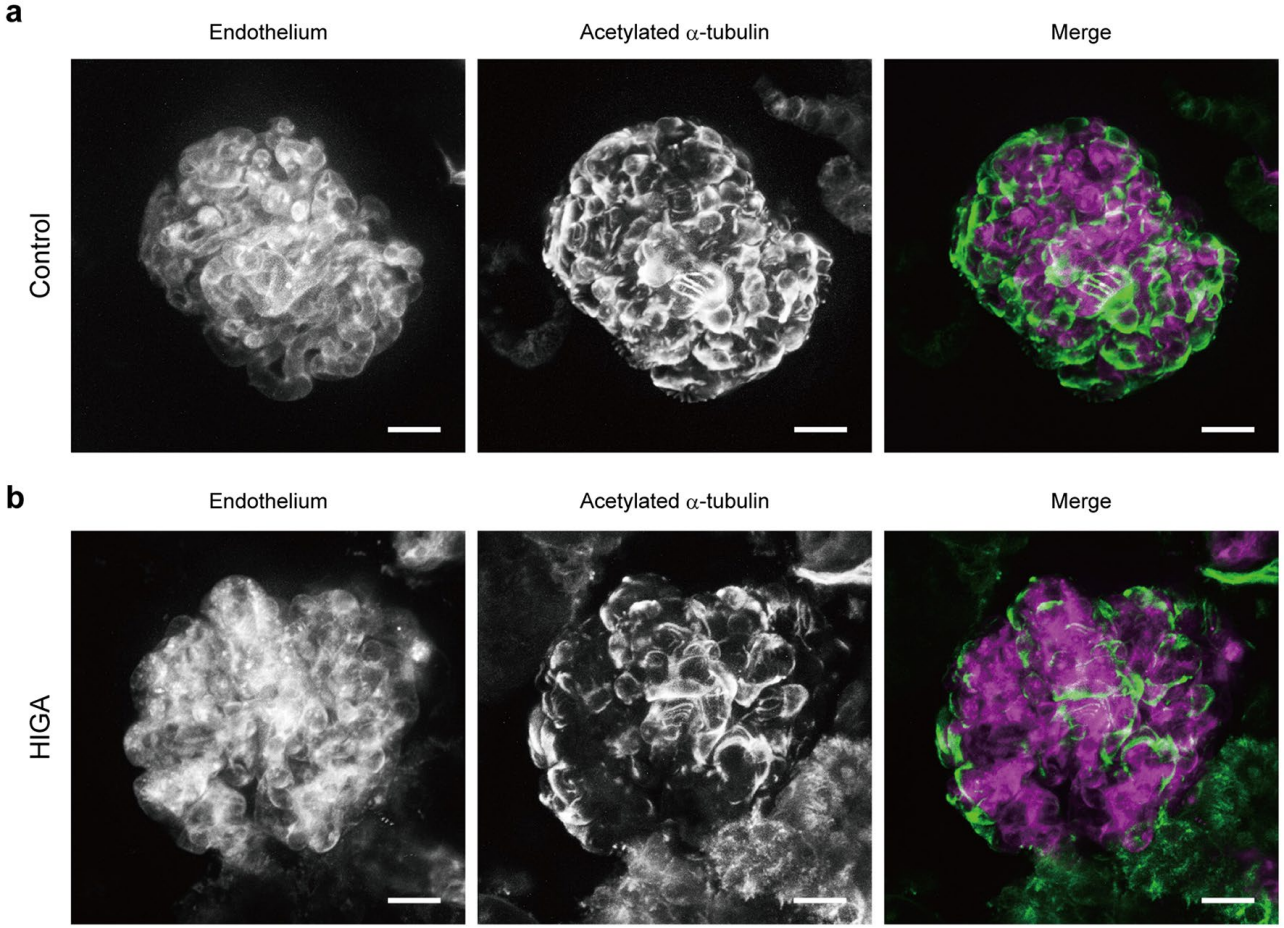
Two-photon microscopy of glomeruli in HIGA mice shows reduced major processes of podocytes. Glomeruli from control BALB/c (**a**) and HIGA (**b**) mice. In the HIGA mouse, five out of six observed glomeruli showed similar abnormalities, while the four BALB/c mouse glomeruli observed were all normal. Images are *XY* maximum projections of 100 optical sections of 1 µm step size. Scale bar = 20 μm. The endothelium of glomerular capillaries is labelled by vessel painting with DiD(C18) (magenta). Cell bodies and major processes of podocytes are labelled by indirect immunostaining using anti-acetylated-α-tubulin antibody as a primary antibody (green).

Finally, we combined vessel painting, immunostaining, and tissue clearing, and performed two-photon microscopy of glomeruli from HIGA mice (Fig. 7). In preliminary experiments, the signal of indirect immunofluorescence of anti-acetylated-α-tubulin antibody disappeared after tissue clearing with OPTIClear. We found that post-fixation with 4% paraformaldehyde following secondary antibody treatment preserves the signal after tissue clearing, possibly due to the cross-linking of antibodies with their epitopes to prevent detachment in the non-physiological tissue clearing solution for refractive index matching. After clearing, the 3D structure of a capillary tuft and associated podocytes in an entire glomerulus could be clearly imaged by two-photon microscopy with SLM-based aberration correction (Supplementary Movies S3 and S4). In control mice, glomerular capillaries labelled with vessel painting were uniformly covered with cell bodies and major processes of podocytes (Fig. 7a), while signs of local podocyte loss were observed in some glomeruli of the HIGA mice (Fig. 7b).

## Discussion

### Introduction of new carbocyanine dyes for vessel painting

To visualise vasculature, several methods, such as genetically encoded fluorescent marker proteins, fluorescently labelled probes, and fluorescent space occupants, have been widely used^1–4^. Among them, vessel painting utilising a carbocyanine dye, DiI, is an easy and cost-effective method^4^. Hydrophobic carbocyanine dyes, such as DiI(C18), label cells by inserting their alkyl chains into the lipid bilayer of the plasma membrane^22^. Those dyes are poorly soluble in an aqueous medium and can be applied in crystalline form for axon tracing experiments^23^. During vessel painting, the aggregation of hydrophobic dyes can result in heterogeneous staining due to the occlusion of capillaries^7,10^. Liposomes are the means of choice to deliver various substances, including hydrophobic ones, to biological systems^24^. It has been reported that liposomes can deliver hydrophobic fluorescent dyes to the plasma membrane of cultured cells through membrane fusion^25^. The liposome-mediated vessel painting technique was devised based on the hypothesis that the reproducibility and staining efficiency of vessel painting would be improved by the prevention of carbocyanine dye aggregation^10^.

In this paper, we have introduced new carbocyanine dyes, DiO(C14), DiD(C18), and DiR(C18), for liposome-mediated vessel painting (Fig. 1). These dyes allow users to select green, deep red, and near-infrared channels for detection, along with the yellow channel of DiI(C12), which should be particularly useful when vessel painting is combined with other probes. Furthermore, DiD(C18) and DiR(C18) have longer excitation/emission wavelengths and thus can extend the attainable imaging depth without the requirement for any change in the procedure^26,27^. An important factor for successful vessel painting is the hydrophobicity of the carbocyanine dye used. A dye that is too hydrophobic aggregates readily in an aqueous environment and results in reduced labelling intensity and reproducibility^10^. Hydrophobicity for the carbocyanine dyes used in this study appears to be affected by three factors. First, when the fluorophore is the same, a dye with shorter alkyl chains is more hydrophilic. For example, DiO(C18) and DiI(C18) were almost-completely and partially insoluble in ethanol at a concentration of 5 mM, respectively, while DiO(C14) and DiI(C12) were readily soluble. Second, when the lengths of alkyl chains are the same, a carbocyanine dye with a longer emission wavelength is more hydrophilic. DiD(C18) and DiR(C18) dissolved easily in ethanol at 5 mM, while DiO(C18) and DiI(C18) did not. This may be explained by the fact that a carbocyanine dye with a longer wavelength has a longer polymethine linker^28^ (Supplementary Fig. S2), which is expected to suppress the stacking of planar ring structures by increasing the number of possible conformational states. The third factor is also related to the possible suppression of stacking between dye molecules. The increased hydrophilicity of Neuro-DiO(C18) over DiO(C18) could be attributed to the addition of bulky tert-butyl groups, which can cause steric hindrance and thus prevent the stacking interactions between the ring structures of the dye^29^. Although Neuro-DiO(C18) showed much better solubility in ethanol than DiO(C18), its liposome solution sometimes caused leakage of the perfusate from the airway. Similar leakage was frequently observed in our previous study with DiI(C18)^10^. The cause of the leakage is likely to be the aggregation of highly hydrophobic dye molecules in the aqueous working solution and the subsequent occlusion of the lung capillaries by those aggregates. Occluded capillaries are vulnerable to rupture due to the increase in local perfusion pressure. In fact, we have observed occlusion and rupture of capillaries in mouse brains perfused with DiI(C18)^10^. Therefore, the hydrophilicity of Neuro-DiO(C18) might not be sufficiently high for liposome-mediated vessel painting. The factors mentioned above may also affect the diffusion rate of a carbocyanine dye in the membranes of axons. Indeed, DiI(C18) and DiD(C18) show faster diffusion rates than DiO(C18)^30^. Less stackable dyes may diffuse faster on two-dimensional membranes because of a reduced tendency to form large multimers.

### Application of vessel painting for various tissues

Liposome-mediated vessel painting is thought to label endothelial cells through membrane fusion between dye-incorporated liposomes and endothelial cells (Supplementary Fig. S1)^10,25^. We demonstrated that the procedure can be used to label vasculature in various organs, owing to the ligand-independent staining principle. In this study, we compared the staining patterns of renal vasculature among liposome-mediated vessel painting and two ligand-dependent probes, tomato lectin and anti-CD31 antibody. Lectins bind to specific sugar chains and do not necessarily label all blood vessels^3^. Indeed, tomato lectin labelled a much smaller fraction of blood vessels than vessel painting in mouse kidneys. We also tested another popular probe for endothelial cells, anti-CD31 antibody. This antibody is frequently used to carry out 3D imaging of blood vessels in various organs^31–33^, but the expression level of CD31 can vary among different endothelial populations^34^. Although the renal vasculature labelling patterns for anti-CD31 antibody and vessel painting were comparable, the signal intensity was much stronger for vessel painting. Furthermore, liposome-mediated vessel painting should be effective for imaging the vascular structure of any species where perfusion is possible, because of its simple staining principle. Since macromolecular probes such as lectins or antibodies have their own advantages, such as fixability or stainability after excision of a tissue, researchers can select a suitable method according to experimental requirements. It should be noted that vessel painting is a perfusion-based method and hence an operation conducted by an individual with less expertise, or the occurrence of occluded vessels, can result in insufficient labelling.

### Comparison of imaging depth of carbocyanine dyes with different wavelengths

Since longer excitation light is less scattered in opaque biological specimens, a fluorescent dye with a longer excitation/emission spectrum is more suitable for deep tissue imaging^26,35^. Although the effect of excitation spectra on imaging depth has been well studied, the experimental data on the effect of emission spectra has been reported only one study with three-photon microscopy of live specimens^35^. Generally, a dye with a longer excitation spectrum also has a longer emission spectrum, making it difficult to separate the effects of the two wavelengths. We noticed that the carbocyanine dyes tested here have secondary absorption bands in a shorter-wavelength spectral region, and two different dyes can be excited when an appropriate excitation wavelength is used. In this study, we simultaneously excited the same concentration of DiO(C14) and DiD(C18) with a two-photon laser and confirmed DiD(C18) is more favorable for deeper imaging of highly scattering fixed tissues^36^. In this experiment, it was possible to simultaneously excite both dyes via two-photon laser excitation at 820 nm, although DiD(C18) has almost no absorption around 400 nm (Fig. 1b). The availability of this two-photon excitation wavelength in DiD(C18) is possibly due to a shift of the absorption band for two-photon excitation^37^.

### Tissue clearing techniques compatible with vessel painting

Tissue clearing techniques that make various tissues and organisms transparent^17–19,38,39^ are powerful tools for 3D visualisation of blood vessel networks. The vasculature is also used as an anatomical landmark in cleared specimens^18,40^. Most tissue clearing protocols have two steps: removal of lipid with a detergent or organic solvent, and refractive index matching. Since DiI and its analogues label plasma membranes by inserting their alkyl chains into the lipid bilayer, vessel painting is incompatible with reagents that extract lipids. Although a few fixable DiI analogues, such as CM-DiI, are commercially available, they are much more expensive than common carbocyanine dyes and are not practical for routine use. Therefore, tissue clearing protocols that utilise organic solvents or detergents cannot be applied to tissues labelled by common carbocyanine dyes. However, some clearing protocols free from lipid extracting reagents have been reported to be compatible with DiI labelling^17,19^. A previous study performed clearing of thick slices of mouse brains with FocusClear after vessel painting^5^. Unfortunately, this proprietary regent is expensive and the composition is not publicised. In the present study, we tested three clearing protocols that are free from detergents or organic solvents and are expected to be compatible with vessel painting: SeeDB^17^, OPTIClear^19^, and Sca*l*eSQ(0)^18^. SeeDB is one of the simplest and the least expensive methods and involves immersing samples in a graded series of fructose solutions^17^. Sca*l*eSQ(0) is a detergent-free variation of Sca*l*eS. It shows excellent preservation of tissue structure and even transmission electron microscopy is possible after tissue clearing^18^. OPTIClear has strong clearing capacity and provides good results even with over-fixed specimens, which are not suited to most aqueous-based clearing methods^19^. Those protocols were originally designed to clear brain tissue and the application to mouse kidney tissue has been reported only for SeeDB^41^. To the best of our knowledge, these three protocols have not been compared so far in the literature. We confirmed that all of the tested protocols can be combined with vessel painting and strongly improve the imaging depth in two-photon microscopy. Among them, OPTIClear provided the best transparency for both brains and kidneys. However, it should be noted that tissue clearing protocols differ not only in terms of achievable transparency but also in terms of cost, time required, procedure complexity, and compatibility with probes. For example, OPTIClear produces excellent transparency, but uses relatively expensive reagents compared to most other tissue clearing methods. The best protocol can differ depending on the research target or means of observation. Compatibility with DiIs was also demonstrated for a recently developed tissue clearing protocol, MACS^42^. This protocol also appears promising for clearing tissues after liposome-mediated vessel painting.

In the present study, we introduced a novel technique to correct the spherical aberration caused by a mismatch between the optimal RI for an objective and the actual RI of an immersion medium. The reduction in image quality (i.e., blurred images and reduced signal-to-noise ratio) generated by such aberrations is more severe for long-working-distance (WD) objectives, which are typically used for two-photon microscopy of cleared specimens, because the effect of the aberration is proportional to the thickness of a medium between the objective and its focus^43^. To image samples in various immersion media with different RIs, several different objectives are generally required. For example, objectives customised for different tissue clearing solutions are commercially available^44^. Another choice is to use an objective with a correction collar. In this case, the physical adjustment of the collar by hand or an automated system is required^45^.

Our aberration-correction technique is based on wavefront modulation by an SLM. An SLM incorporated within a two-photon microscope can electrically correct the deviation of the focal point of an objective immersed in a medium with non-optimal RI value to reduce spherical aberration. It allows the adoption of a single immersion lens for a variety of media with a wide range of RI, from water (RI = 1.33) to various tissue clearing solutions (RI = ~ 1.50), and even epoxy resin (RI = 1.59). Furthermore, our technique works if the RI of a medium is known and therefore does not require feedback from a wavefront sensor for adaptive optical aberration correction^46^. This makes the optical system simpler and less expensive. With our aberration-correction technique, we used a single water-immersion objective to successfully acquire high-quality images from mouse brain and kidney tissues immersed in high-RI clearing solutions.

### Glomeruli can be labelled with various probes after vessel painting

Vessel painting has been mainly used for single staining. To make the technique more versatile, we attempted to combine it with other histological methods, which are applied to the tissue after vessel painting. As mentioned above, permeabilisation with a detergent cannot be applied to tissues labelled with a lipophilic carbocyanine dye used for vessel painting. However, we expected that at least the near-surface volume of the tissue slices could be labelled by other probes, such as an antibody, since fixed dead cells lose their selective permeability for macromolecules.

In the present study, we selected the kidney as a model organ for two reasons: 1) it is a porous organ and hence is expected to be favourable for probe penetration, and 2) multi-labelling and volume imaging of blood vessels and associated structures in renal glomeruli are helpful for understanding the pathology of many kidney diseases. The glomerulus is a unit for ultrafiltration composed of the glomerular capillary tuft, GBM, and podocytes. As expected, entire glomeruli exposed to the surface of renal slices could be labelled with small probes and even antibodies without specific permeabilisation steps in only a 1 h incubation period. The omission of thin sectioning enabled the imaging of 3D glomerular structures that had not been mechanically disturbed.

To our surprise, we found that the imaging of the intact glomeruli resolved podocyte foot processes in some detail. Foot processes are generally believed to be unresolvable via diffraction-limited optics, and therefore they have been the subject of various super-resolution microscopy studies^47,48^. The resolution obtained in the present study should be attributed to the use of a small probe (phalloidin), the omission of thin-sectioning (which disrupts fine structures), and the use of high-NA objectives or, or in combination with, the aberration correction technique. Although electron or super-resolution microscopies have, of course, better resolution, our strategy does not require specialised skills or equipment and can be performed as a simple extension of conventional histological analyses. On the larger scale, the combination of the fluorescent labelling, tissue clearing, and light-sheet microscopy allows the 3D visualisation of the vasculature of an entire mouse organ^49,50^. However, this approach is not suitable for the detailed observation of individual glomeruli within an entire organ because of the trade-off between resolution and the size of image files and/or because of difficulties in the immunostaining of large specimens. Our approach is an easy and rapid option for 3D imaging of glomeruli, or any structures associated with blood vessels, at subcellular resolution and can fill the gap between super-resolution and large volume 3D imaging strategies.

### Pathological application of liposome-mediated vessel painting

To demonstrate the advantage of vessel painting in pathological analyses, we performed imaging experiments with kidneys from HIGA mice, an IgA nephropathy model mouse strain. This strain was produced by selective breeding of an earlier IgA nephropathy model, the ddY strain, with higher serum IgA level^14^ to increase the incidence of glomerulonephritis with IgA deposition in the ddY strain^51^. HIGA mice show a rapid increase in serum IgA level at between 10 and 25 weeks of age. Proteinuria occurs in 10% of them at 10 weeks of age and the incidence and severity increase with age^13,14^. Morphologically, transmission electron microscopy has revealed that the local thickening of the GBM (GBM nodule) increases markedly in this strain^13,15^. In the present study, GBM nodules were observed as abnormal foam-like structures of foot processes (Supplementary Fig. S6, Supplementary movies S1 and S2). The abnormal structures were confirmed as the protrusions of GBM by 3D imaging of glomeruli involving the labelling of three layers of the ultrafiltration unit (Fig. 6 and Supplementary Fig. S6). This is the first observation of this minute lesion via light microscopy. Although the morphology and age-related occurrence of GBM nodules are well described by TEM in the ddY strain from which the HIGA strain originated, in Ref. 20, the molecular mechanism of their formation and the pathological value of their detection remains unclear. The study reported that GBM nodules are homogeneous structures with similar density to the lamina densa of GBM and suggested that they were formed by an abnormal metabolism of connective tissue. This observation is consistent with our imaging result that anti-collagen IV antibody labelled the nodules homogeneously. We also observed suspected penetration of the liposome–carbocyanine dye solution through the ultrafiltration barrier, as demonstrated by the staining of podocytes in HIGA mice (Supplementary Fig. S7). This result might reflect the fact that proteinuria is one of the pathologies of HIGA mice. Although it should be further validated in the future, liposome-mediated vessel painting might allow easy detection of leaky glomeruli.

In HIGA mice, severe IgA deposition is also reported in the mesangial area as early as 25 weeks of age, as well as mild deposition of IgG and C3^52,53^. They have only been observed in conventional tissue sections and their localisations have not been demonstrated in 3D at subcellular resolution. It is interesting to perform an imaging study of those abnormal depositions in IgA model mice, in the context of the entire glomerulus, using our approach.

Finally, we combined liposome-mediated vessel painting with all the histochemical methods tested in this study, that is, a newly introduced carbocyanine dye, multi-labelling, and tissue clearing, for the pathological analysis of the glomeruli of HIGA mice. Immunostaining of tubulin and tissue clearing with OPTIClear after vessel painting allowed the visualisation of capillary tufts and surrounding podocytes in the entire glomerulus. We found reduced coverage of a capillary tuft with podocytes in some of the HIGA mouse glomeruli. Since microtubules form the major cytoskeleton of podocyte cell bodies and major processes, the loss of their signal appears to indicate the loss of podocytes. Since many glomerular diseases accompany the loss of podocytes, this approach would be useful in evaluating pathological changes of glomeruli.

In conclusion, liposome-mediated vessel painting can be easily combined with other histochemical methods. It serves as an easy and cost-effective technique to visualise the 3D vasculature and associated structures of small mammals in healthy and diseased conditions.

## Methods

### Animals

Twenty-six C57BL/6 J (8–10 weeks old), six HIGA (25–30 weeks old), and six BALB/c (25–30 weeks old) female mice were obtained from Japan SLC (Hamamatsu, Japan). They were kept under the specific-pathogen-free condition on a 12-h dark/light cycle with food and water ad libitum until use. All animal experiments were approved by the Institutional Animal Care and Use Committee of Hamamatsu University School of Medicine (Permission number: 2018012) and were performed in accordance with relevant guidelines and regulations.

### Reagents

The following reagents were used: DiO(C14), Neuro-DiO(C18), DiR(C18) (PromoCell, Heidelberg, Germany; #PK-CA707-60012, #PK-CA707-60015, and #PK-CA707-60017, respectively); DiO(C18), DiI(C12), and goat anti-mouse IgG antibody, Alexa 568 conjugate (Thermo Fisher Scientific, Waltham, MA, USA; #D275, #D383, and #A-11004 respectively); DiD(C18) (Biotium, Fremont, CA, USA; #60014); 1 mg/mL medetomidine hydrochloride (Domitor; Zenoaq, Fukushima, Japan), 5 mg/mL midazolam (Sandoz, Tokyo, Japan), and 5 mg/mL butorphanol tartrate (Meiji Seika Pharma, Tokyo, Japan); Coatsome-EL-01-N (Yuka Sangyo, Tokyo, Japan); Neutral Niosome (Nanovex Biotechnologies, Madrid, Spain; #PNS-NN); DyLight 594 labelled *Lycopersicon esculentum* (tomato) lectin (Vector Laboratories, Burlingame, CA, USA; #DL-1177); rhodamine-phalloidin (Acti-stain 535; Cytoskeleton, Denver, CO, USA; #PHDR1); anti-α-tubulin monoclonal antibody (mAb), Alexa 488 conjugate (DM1A-Alexa 488; Santa Cruz Biotechnology, Dallas, TX, USA; #sc-32293 AF488); anti-acetylated tubulin mAb, clone 6-11B-1 (Sigma-Aldrich, St. Louis, MO, USA; #T7451); anti-mouse CD31 antibody, Alexa 488 conjugate, clone MEC13.3 (BioLegend, San Diego, CA, USA; #102514); 1 mg/mL 4′,6-diamidino-2-phenylindole (DAPI) solution (Dojindo Laboratories, Kumamoto, Japan; #340–07971); α-thioglycerol and iohxol (Tokyo Chemical Industry, Tokyo, Japan; S0374 and I0903, respectively); bovine serum albumin (Katayama Chemical, Osaka, Japan; #01–2030). Other reagents were purchased from FUJIFILM Wako Pure Chemical Corporation (Osaka, Japan).

### Preparation of fluorescently labelled suncus mAb for anti-type IV collagen antibody

Suncus mAb for type IV collagen (STF31)^21^ was purified by MEP HyperCel™ Chromatography (Nihon Pall Ltd., Tokyo, Japan). Fluorescent labelling of the mAb with fluorescein isothiocyanate (FITC) was performed as previously described^54^.

### Liposome-mediated vessel painting

Dye stock solutions were prepared by dissolving carbocyanine dyes in ethanol at a concentration of 5 mM. They were stored at room temperature (RT) in the dark. Working solution (50 μM dye, 1 mg/mL neutral liposome in PBS) was prepared just before vessel painting. Since Coatosme-EL-10-N, a neutral liposome used in the original protocol^10^, was discontinued, we tested other liposomes and found that Neutral Niosome (Nanovex Biotechnologies) provides comparable results. First, Neutral Niosome was added to PBS (10 mL per mouse) at a concentration of 1 mg/mL and incubated at 60 °C for approximately 30 min as per the manufacturer’s instructions. Then, a stock dye solution was added at the final concentration of 50 μM (100 μL of a dye stock solution per 10 mL of liposome solution) and the mixture was immediately vortexed and sonicated. An injection device was constructed with a three-way stopcock and two 10 mL syringes, and a 25G butterfly needle (Supplementary Fig. S8).

Mice were anaesthetised by intraperitoneal injection of 10 μL/g body weight of mixed anaesthetic (75 µg/mL medetomidine hydrochloride, 0.4 mg/mL midazolam, 0.5 mg/mL butorphanol tartrate in saline)^55^. For transcardial perfusion, the needle of the injection device was inserted into the left ventricle, and the right atrium was cut for drainage. Then, 10 mL of the dye working solution and 10 mL of 4% paraformaldehyde in 0.1 M PB (pH 7.4) were sequentially injected at a flow rate of ~ 2–3 mL/min. After perfusion, the organs of interest were isolated and further fixed in the same fixative for 1‒2 h at RT or overnight at 4 °C on a shaker. Brains and lungs were processed without further dissection. Kidneys and livers were manually cut with razor blades into 1–2 mm thick slices and the intestine was cut into short segments and opened with dissecting scissors before immersion fixation. For double vessel painting with DiO(C14) and DiD(C18), 10 mL working solutions of DiO(C14) and DiD(C18) were prepared separately. They were then mixed and 20 mL of the dye mixture solution was perfused.

### Vascular staining with tomato lectin and anti-CD31 antibody

Tomato lectin staining was performed as previously reported^3^. Briefly, mice were anaesthetised as described above and a mixture of 100 μL of fluorescently labelled tomato lectin and 100 μL of saline was injected into the left ventricle with a 30G needle. After the heart was allowed to beat for about 1 min, 10 mL of PBS and 10 mL of 4% PFA in 0.1 M PB (pH 7.4) were manually perfused as liposome-mediated vessel painting. For immunostaining, 15 µg of anti-CD31 antibody was diluted to a total volume of 100 µL with saline and retro-orbitally injected^55^. After 10 min, perfusion fixation was performed.

### Spectroscopy

Carbocyanine dyes were dissolved in ethanol at concentrations of 2 mM and 10 mM. The absorbance of each dye was measured with a U-3500 spectrometer (Hitachi, Tokyo, Japan).

### Tissue clearing

Fixed brain and kidney were washed three times with PBS, for 10 min each, and subjected to one of the following tissue clearing protocols: SeeDB^17^, OPTIClear^19^, and a detergent-free form of Sca*l*eS [Sca*l*eSQ(0)]^18^. For SeeDB, samples were incubated in a graded series of fructose solutions [20, 40, 60, and 80% (w/v) fructose in ddH_2_O] for at least 3 h each, and then in SeeDB solution [80.2% (w/w) fructose, 0.5% (v/w) α-thioglycerol in ddH_2_O] overnight. For Sca*l*eSQ(0), samples were incubated in Sca*l*eSQ(0) solution [9.1 M urea, 22.5% (w/v) D-( −)-sorbitol, in ddH_2_O (for 100 mL: 54.7 g urea and 22.5 g D-( −)-sorbitol)] at 37 °C and then in Sca*l*eS4(0) solution [4 M urea, 40% (w/v) D-( −)-sorbitol, 10% (w/v) glycerol, and 25% (v/v) DMSO in ddH_2_O] overnight at RT. For OPTIClear, samples were incubated in OPTIClear solution [20% (w/v) N-methylglucamine, 32% (w/v) iohexol, and 20.48% (v/v) 2,2′-thiodiethanol in ddH_2_O, pH 7–8, adjusted with HCl] at least 4 h at RT. In all steps, samples were gently agitated on a shaker.

### Confocal microscopy

Samples were placed in a glass-bottomed dish and imaged with an SP8 microscope (Leica, Wetzlar, Germany) equipped with HC PL APO CS2 20 × /0.75 DRY and HC PL APO CS2 63 × /1.40 oil objectives (Leica). The pinhole was closed to its minimum diameter during all observations. Laser power at the sample surface was set, in each experiment, to the maximum possible value that did not result in signal saturation and was kept constant throughout each imaging session.

### Two-photon microscopy and aberration correction

Samples were attached to the bottom of a 60-mm plastic dish with cyanoacrylate glue and immersed in an imaging medium. Images were acquired by using a custom-made setup, as previously described^57,58^. A water immersion objective (XLUMPLFLN20XW 20 × /1.0 Olympus) was used for all the experiments performed in this study. We incorporated an SLM (X10468, Hamamatsu Photonics K.K.) into the two-photon microscope to allow electrical correction of aberrations^12^. We predicted a wavefront aberration according to the following equation and cancelled it by applying a reverse wavefront:

$${\Phi }\left( \rho \right) = - \left( {\frac{{2\pi {\text{WD}}}}{\lambda }} \right)\left( {\left( {1 + \eta } \right)\left( {\sqrt {n_{2}^{2} - \left( {{\text{NA}}\rho } \right)^{2} } } \right) - \left( {\sqrt {n_{1}^{2} - \left( {{\text{NA}}\rho } \right)^{2} } } \right)} \right),$$

where *λ* is the wavelength of the excitation beam, *ρ* is the normalised pupil radius, *η* is the factor for changing the depth of the focal spot, *n*_1_ and *n*_2_ are the RIs of water and tissue clearing solutions, respectively, and NA and WD are the numerical aperture and the working distance of the objective used.

DiO, DiI, DiD, and DiR were excited with excitation wavelengths of 915 nm, 880 nm, 850 nm, and 915 nm, respectively. To compare the DiO and DiD imaging depths, the excitation wavelength was set to 820 nm, and the laser power was adjusted at the surface of the sample so as to ensure that the signal was not saturated; the power was kept constant during each imaging session.

### Multi-labelling of the kidney

After liposome-mediated vessel painting, the kidneys were manually cut using a razor into 1–2 mm thick slices, washed three times for 10 min each with PBS and incubated in blocking solution (5% bovine serum albumin in PBS) for 1 h. For confocal microscopy, the samples were labelled in the blocking solution containing the following fluorescent labels (300–500 μL for a bisected half of a kidney), for 1 h, in various combinations: rhodamine-phalloidin (1:100), the anti-α-tubulin mAb DM1A-Alexa488 (1:100), and DAPI (1 μg/mL). The slices were washed three times for 10 min each with PBS and were imaged as described above. For two-photon microscopy, the samples were incubated in a blocking solution containing anti-acetylated α-tubulin mAb (1:100) for 1 h and washed with PBS three times (10 min each). Then, they were labelled with anti-mouse IgG Alexa 568 conjugate (1:100) for 1 h, washed three times with PBS, and fixed using 4% PFA in 0.1 M PB (pH 7.4) for 1 h. Samples were then washed two times with PBS, transferred to PBS containing DAPI (1 μg/mL), and incubated for 30 min. Finally, they were attached to the bottom of a 60-mm plastic dish and incubated with OPTIClear solution for 3 h at 37 °C and then for at least 30 min at RT. All steps were performed at RT (except for the incubation in OPTIClear solution) with gentle agitation.

### Software

Spectral data were handled with Igor Pro software (WaveMetrics, Lake Oswego, OR, USA). Confocal and two-photon images were acquired via LAS X software (Leica, Wetzlar, Germany) and custom-made software, respectively. Optical section images were manipulated with ImageJ software. Chemical structures were drawn with the ACD/ChemSketch freeware (ACD/Labs, Toronto, Canada). Figures were prepared with Adobe Photoshop 2019 and Adobe Illustrator 2019 (Adobe, San Jose, CA, USA).

## Supplementary information

Supplementary Figures

Supplementary Movie 1

Supplementary Movie 2

Supplementary Movie 3

Supplementary Movie 4

## Data Availability

Data are available from the corresponding author on reasonable request.
