# Supplementary material for: Pathological application of carbocyanine dye-based multicolour imaging of vasculature and associated structures
Source: Sci Rep. 2020 Jul 28;10:12613. doi: 10.1038/s41598-020-69394-0 (PMC7387484; doi:10.1038/s41598-020-69394-0)
Supplement: Supplementary file 1 — Supplementary Figures [file 41598_2020_69394_MOESM1_ESM.pdf]

Supplementary Information

Pathological application of carbocyanine dye-based multi-colour imaging of vasculature and associated structures

Alu Konno[1], Naoya Matsumoto[2], Yasuko Tomono[3], Shigetoshi Okazaki[1]

[1]HAMAMATSU BioPhotonics Innovation Chair, Institute for Medical Photonics Research, Preeminent Medical Photonics Education & Research Center, Hamamatsu University School of Medicine, Hamamatsu, Japan

[2]Central Research Laboratory, Hamamatsu Photonics K.K., Hamamatsu, Japan

[3]Division of Molecular and Cell Biology, Shigei Medical Research Institute, Okayama, Japan

Correspondence & Lead Contact: Shigetoshi Okazaki. E-mail: okazaki@hama-med.ac.jp

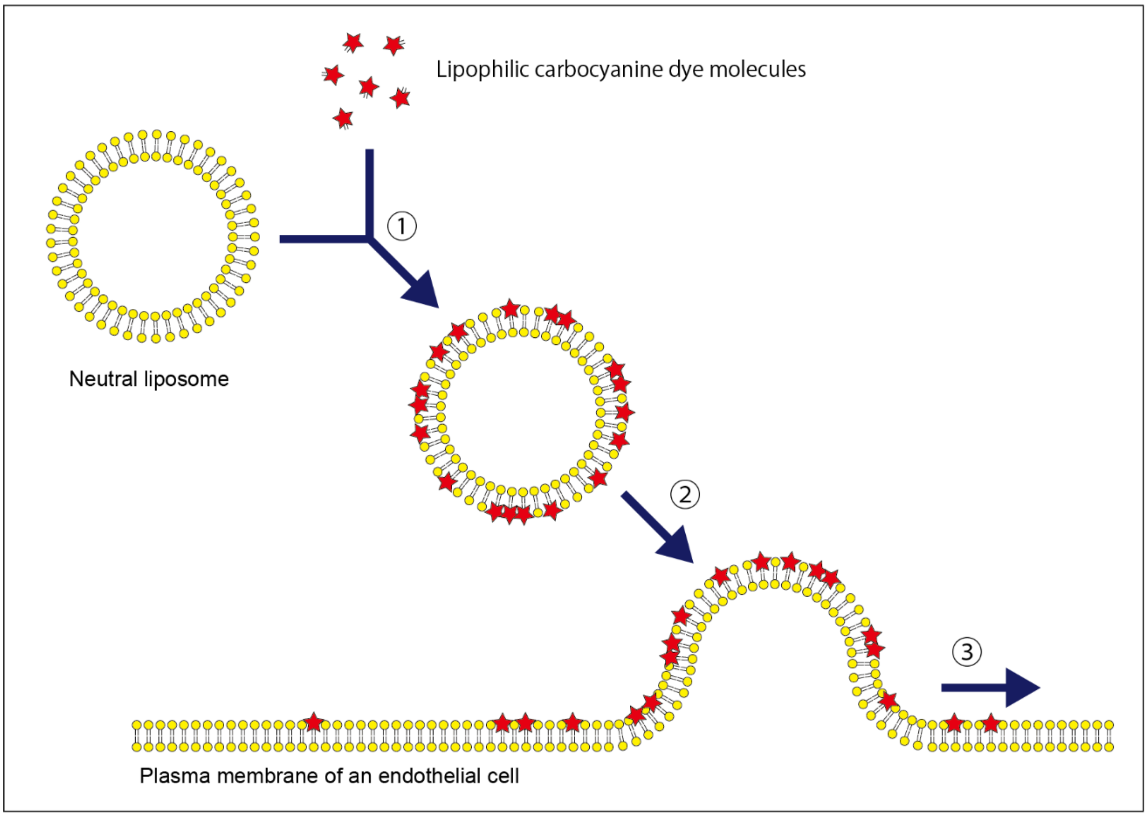

Supplementary Figure S1. Expected molecular mechanism of liposome-mediated vessel painting. 1) Alkyl chains of lipophilic carbocyanine dye molecules are inserted into the liposomal membrane. 2) Liposomes with inserted dye molecules fuse with the plasma membrane of endothelial cells upon contact during perfusion. 3) Dye molecules diffuse laterally, labelling the plasma membrane.

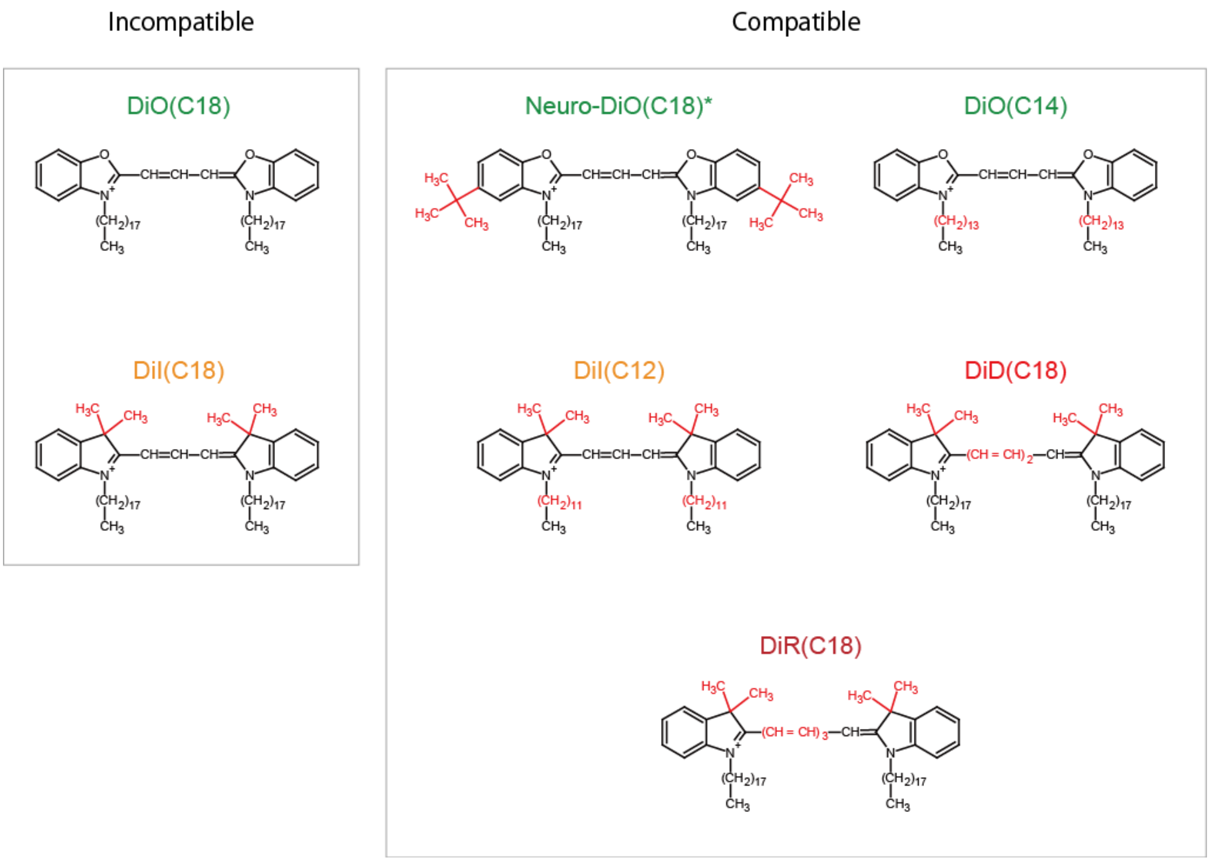

Supplementary Figure S2. Compatibility of DiIs and their analogues with liposome-mediated vessel painting. At a concentration of 50 mM in ethanol, DiO(C18) and DiI(C18) are almost completely and incompletely soluble, respectively. Other dyes are readily soluble and compatible with liposome-mediated vessel painting. *Neuro-DiO(C18) occasionally causes leakage of perfusate from an airway during infusion of working solution. Functional groups shown in red have possible positive effects on hydrophilicity and, therefore, on compatibility with vessel painting.

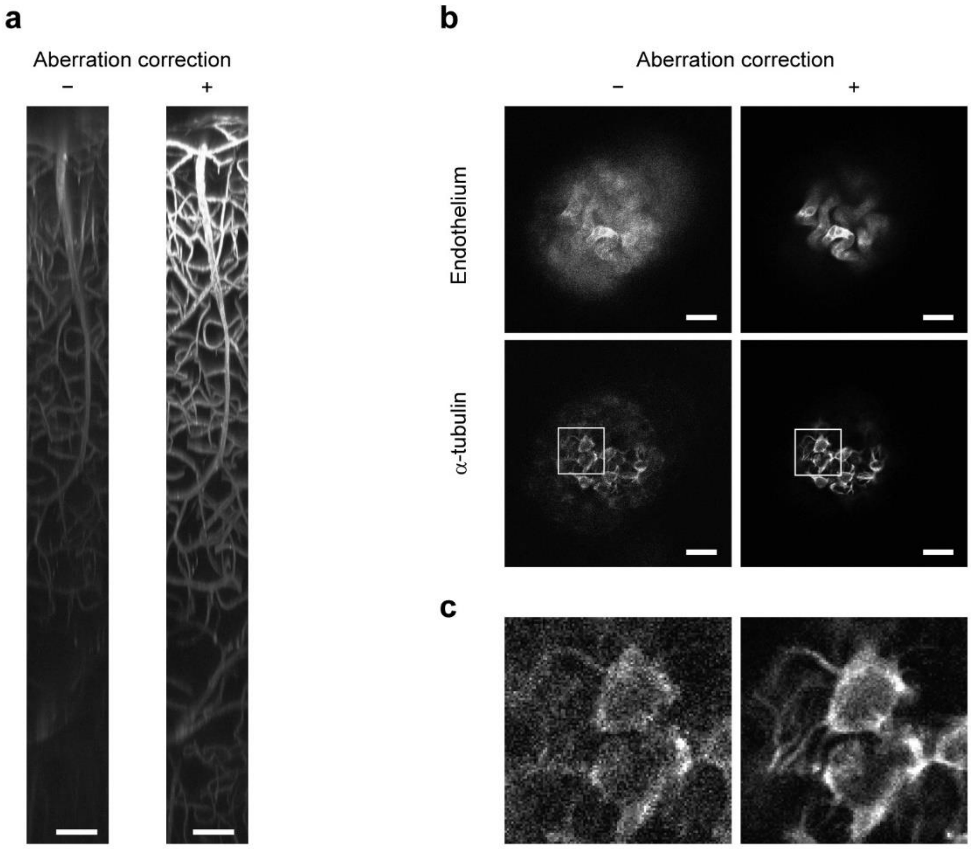

Supplementary Figure S3. The novel aberration-correction technique improves both signal intensity and signal-to-noise ratio. (**a**) *XZ* projection of a brain after liposome-mediated vessel painting with DiR(C18) and tissue clearing with SeeDB. (**b, c**) Optical sections of a glomerulus labelled by liposome-mediated vessel painting with DiR(C18) and fluorescently-labelled anti-α-tubulin antibody and then cleared with OPTIClear. The brightness of the images was standardised. Panels in (**c**) are magnified images from the lower panels in (**b**). Scale bars = 100 μm (**a**; the height of the columns is 1800 μm) and 20 μm (**b**).

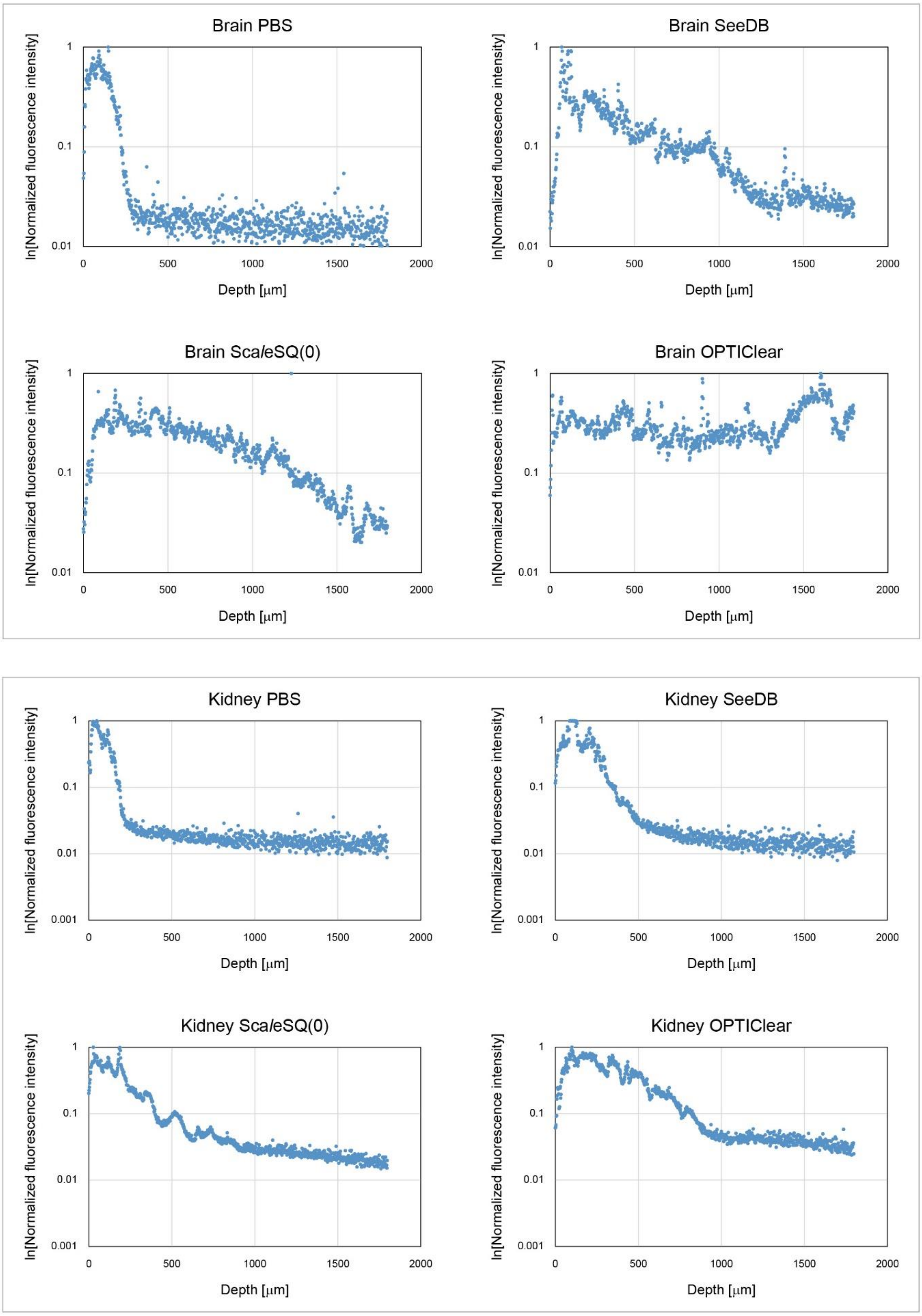

Supplementary Figure S4. Comparison of signal attenuation rates in deep brain and kidney tissues cleared after vessel painting with DiR(C18). The raw data is the same as that shown in Figure 4.

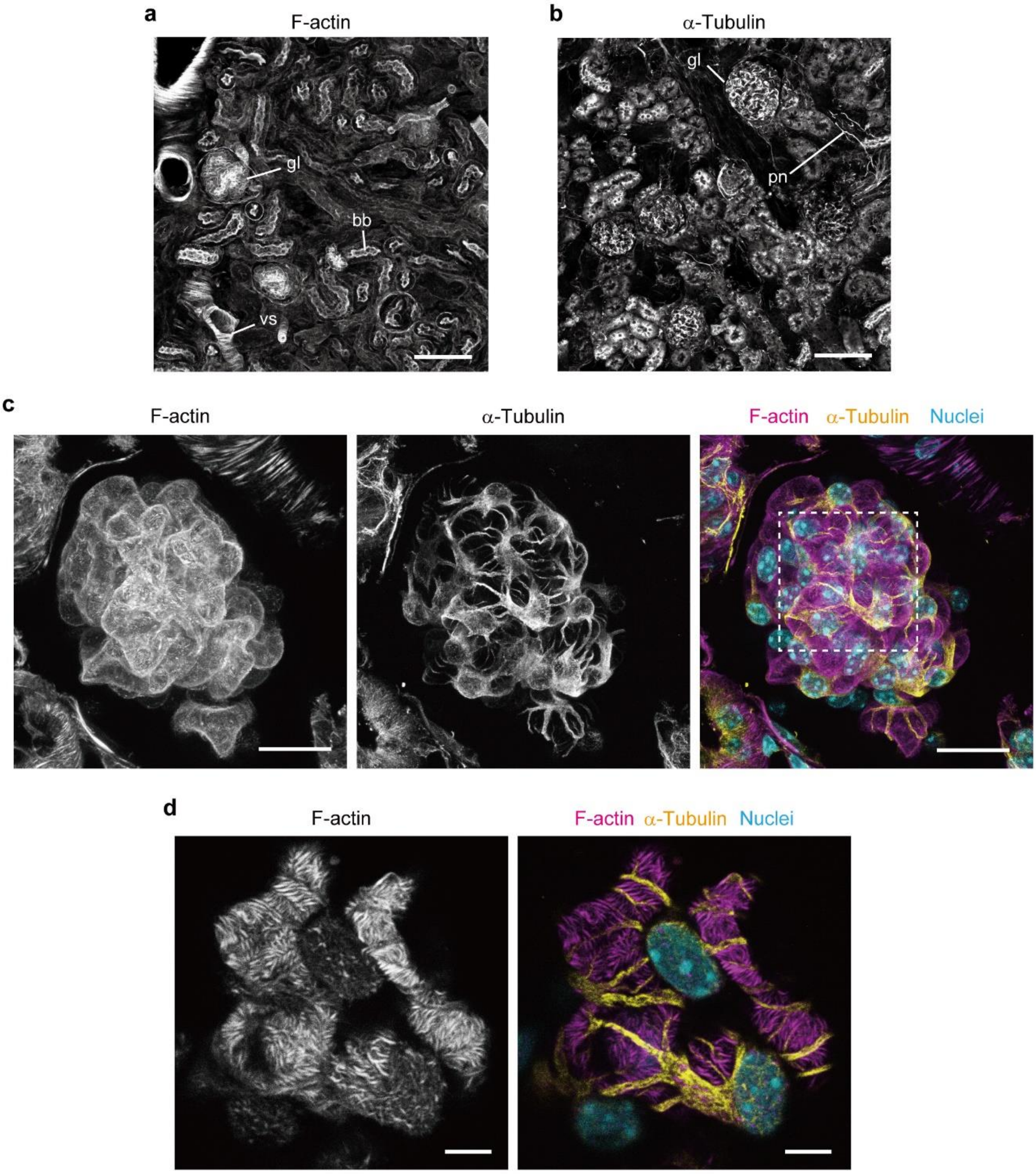

Supplementary Figure S5. Exploration of probes that can visualise general histology of the kidney and do not require permeabilisation with detergents. *XY* maximum projections of confocal images of the surface of renal slices labelled with (**a**) fluorescently labelled phalloidin (Acti-stain 535). (60 optical sections; step size of 1 μm), or (**b**) anti-α-tubulin monoclonal antibody (DM1A-Alexa 488) (99 optical sections; step size of 1 μm). (**c**) Z-projection of confocal images of an intact glomerulus (42 optical sections; step size of 0.5 μm).

The area surrounded by the dashed line is shown magnified in (**d**). (**d**) *Z*-projection of three consecutive optical sections at the surface of the glomerulus shown in (**c**). Some foot processes are visible. bb, Brush border of proximal tubule; gl, glomerulus; pn, peripheral nerve fibre; vs, vascular smooth muscle. Scale bars indicate 100 μm (**a, b**), 20 μm (**c**), and 5 μm (**d**). $N = 2$; representative images from among 19 observations.

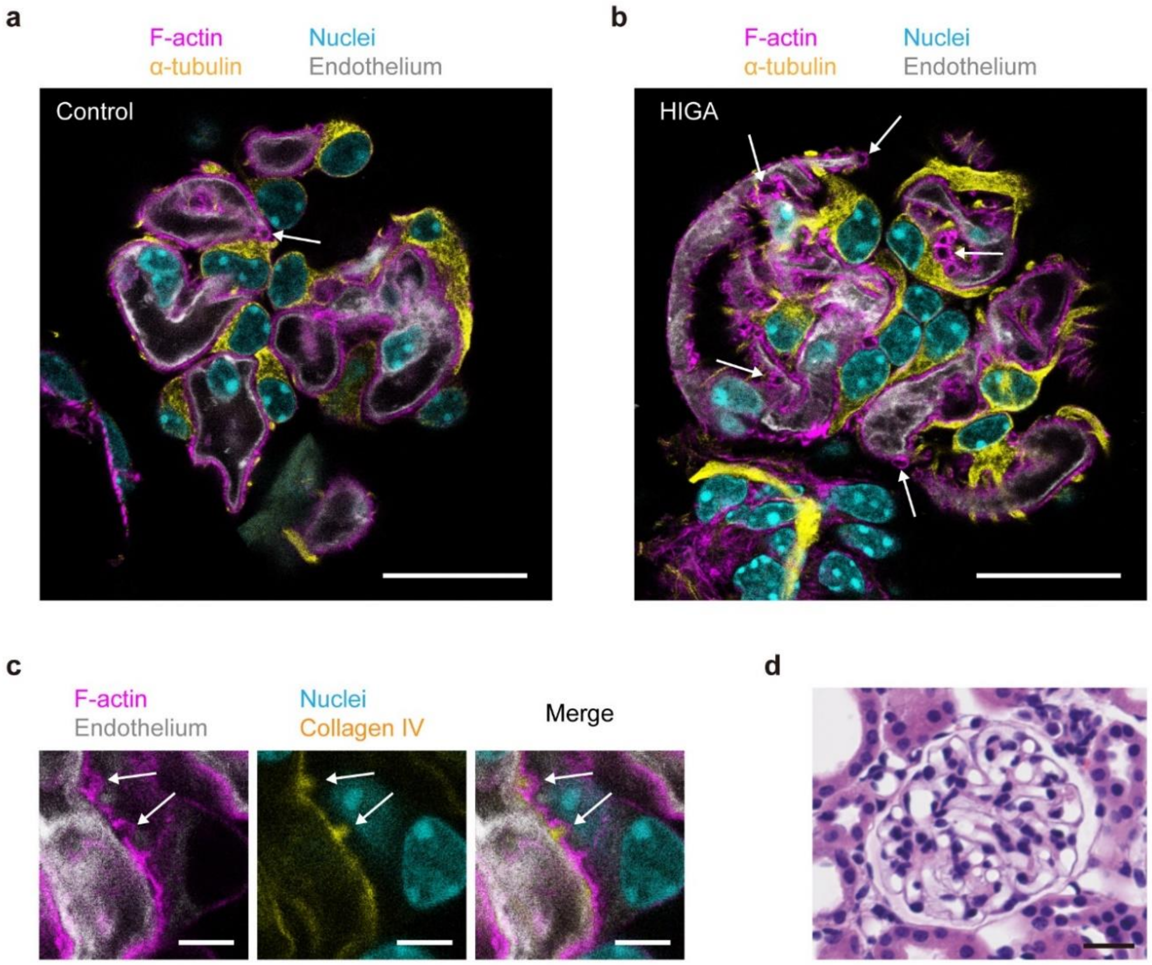

Supplementary Figure S6. Confocal microscopy of vessel-painted glomeruli. (**a, b**) Quadruple staining of a 25-week-old HIGA mouse (**b**) and an age-matched BALB/c mouse (**a**) by vessel painting with DiD(C18) (endothelium, grey), fluorescently labelled phalloidin (f-actin, magenta), anti-α-tubulin mAb (yellow), and DAPI (nuclei, cyan). Arrows indicate some of the large GBM nodules. N = 2; representative images from among 28 (HIGA) and 22 (BALB/c) observations. (**c**) A closer look at the foam-like structures in a glomerulus of the 29-week-old HIGA mouse. Anti-collagen IV antibody staining reveals that the structures are outwardly swelled glomerular basement membrane. Arrows indicate GBM nodules. N = 2; representative images from among 9 observations. (d) A representative image of a glomerulus from a HIGA mouse stained with haematoxylin and eosin. Scale bars indicate 20 μm (**a**, **b**, and **d**) and 5 μm (**c**).

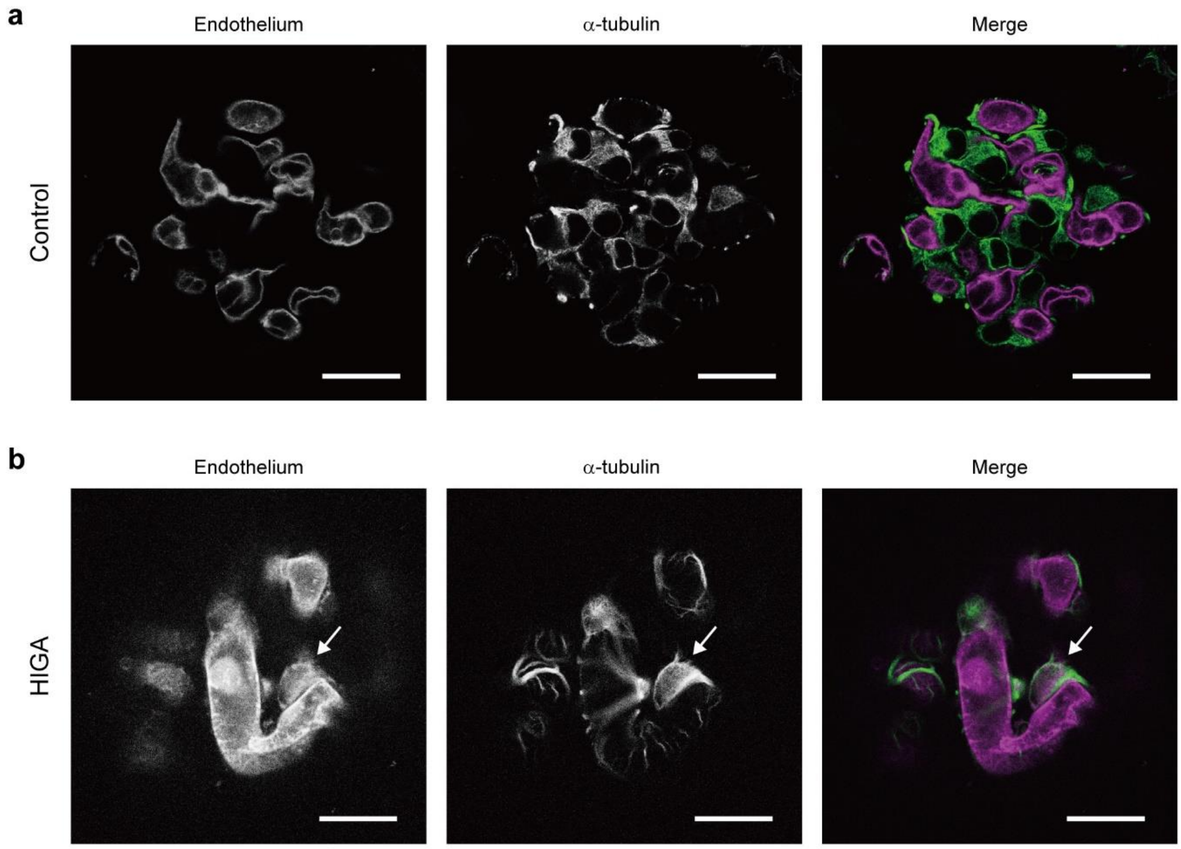

Supplementary Figure S7. Possible leakage of liposomes through the ultrafiltration barrier of glomeruli in HIGA mice. Representative confocal optical sections of glomeruli from age-matched (**a**) BALB/c and (**b**) HIGA mice. Endothelium and podocytes are labelled by liposome-mediated vessel painting with DiD(C18), and anti-α-tubulin antibody, respectively. Arrows indicate the possible leakage site at which a podocyte is positive with DiD(C18). DiD(C18) positive podocytes were found in 10 out of 18 glomeruli in HIGA mice. No similar pattern was observed among 20 glomeruli observed in controls. Scale bar indicates 20 μm; N = 2 for both HIGA and control BALB/c mice.

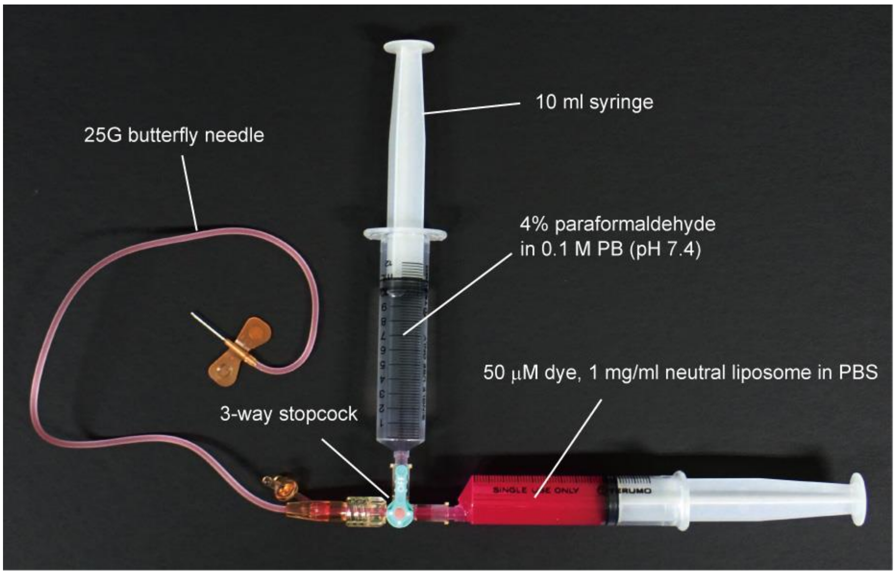

Supplementary Figure S8. Injection device for liposome-mediated vessel painting.

Supplementary Movies S1 and S2. Serial optical sections (*z* stack) of the glomeruli of a control BALB/c mouse (Movie 1) and a HIGA mouse (Movie 2) shown in Figures 6b and 6d, respectively. The left top panel is the merged image of vessel painting with DiD(C18) (endothelium: cyan), phalloidin (foot processes: magenta, and anti-collagen IV antibody (GBM: yellow). (*z*-step size = 0.4 μm; scale bar = 5 μm.)

Supplementary Movie S3. Aberration-corrected (left) and uncorrected (right) serial optical sections of a single glomerulus. Vasculature and podocytes were labelled with DiD(C18) (magenta) and anti-acetylated α-tubulin antibody (green), respectively. (*z*-step size = 0.75 μm; scale bar = 20 μm.)

Supplementary Movie S4. Serial optical sections of the glomeruli of the control BALB/c (left) and HIGA (right) mice shown in Figures 7a and 7b, respectively. Vasculature and podocytes are labelled with DiD(C18) (magenta) and anti-acetylated α-tubulin antibody (green), respectively. (*z*-step size = 0.75 μm; scale bar = 20 μm.)
